# Colonic Lymphangiomatosis

**DOI:** 10.7759/cureus.39085

**Published:** 2023-05-16

**Authors:** Adil S Mir, William F Abel, David P Lebel

**Affiliations:** 1 Gastroenterology, Virginia Tech Carilion School of Medicine, Roanoke, USA; 2 Internal Medicine, Carilion Clinic, Roanoke, USA; 3 Pathology, Dominion Pathology Associates, Roanoke, USA; 4 Basic Science Education, Virginia Tech Carilion School of Medicine, Roanoke, USA

**Keywords:** primary intestinal lymphangiectasia, lymphangiectasia, lymph, sigmoid, sigmoid colon, sigmoid perforation, intestinal perforation, micro-perforation, non-cancer, colonic mass

## Abstract

Lymphangiomas are benign malformations of the lymphatic vessels which can be primary or secondary in etiology. Colonic involvement is rare, and the diagnosis is mostly incidental. Sometimes, the initial endoscopic appearance can be misleading. We present a case of colonic lymphangiomatosis presenting with free air under the diaphragm requiring surgical removal of the involved portion of the colon. The diagnosis was confirmed by the pathology of the resected specimen and its correlation with prior clinical information. The patient recovered well with an uneventful postoperative course and follow-up. This case demonstrates a rare complication of colonic lymphangiomatosis prompting definitive treatment by surgical resection.

## Introduction

Multiple colonic lymphangiomas, or colonic lymphangiomatosis (CL), are a rare clinical entity and are mostly diagnosed incidentally [1]. With the increased use of colonoscopy for screening and therapeutic purposes, CL is identified and reported more often. Although mostly an incidental finding on endoscopic exams, it is rare for CL to present with clinical signs and/or symptoms. We present a case of a symptomatic patient in whom the diagnosis of CL involving a significant area of the sigmoid colon was prompted by the presence of free intraperitoneal air, which was not amenable to endoscopic removal. This article was previously presented as a poster at the Annual American College of Gastroenterology Meeting in Charlotte, USA, on October 25, 2022 (E0134).

## Case presentation

A 73-year-old male with a history of diabetes mellitus and chronic kidney disease underwent computed tomography (CT) abdomen and pelvis with IV contrast for the evaluation of microscopic hematuria of nearly three months duration, which showed the incidental finding of free intraperitoneal air. At this time, the patient did not have abdominal pain, fever, or any signs of sepsis. The patient had a history of colonoscopy for colon cancer screening a few weeks prior to this presentation which showed a "large concentration of polyps" in the sigmoid colon, not amenable for endoscopic removal. The patient had plans for a sigmoidectomy in the coming weeks. However, nearly four weeks after the initial CT scan, the patient developed abdominal pain and nausea, and labs showed worsening leukocytosis 17 K/µL (reference range: 4.0-10.5 K/µL) from the previous level of 9.9 K/µL three months ago. Repeat CT abdomen at this time revealed moderate free intraperitoneal air, worse as compared to previous, and innumerable colonic mucosal cysts of the very redundant sigmoid colon. The patient underwent exploratory laparotomy, and although no frank overt perforation was seen, the sigmoid colon exhibited soft-tissue fullness during the gross intraoperative examination. Given the planned sigmoidectomy given the prior endoscopic findings, a partial sigmoid colectomy with primary anastomosis was performed.

**Figure 1:**
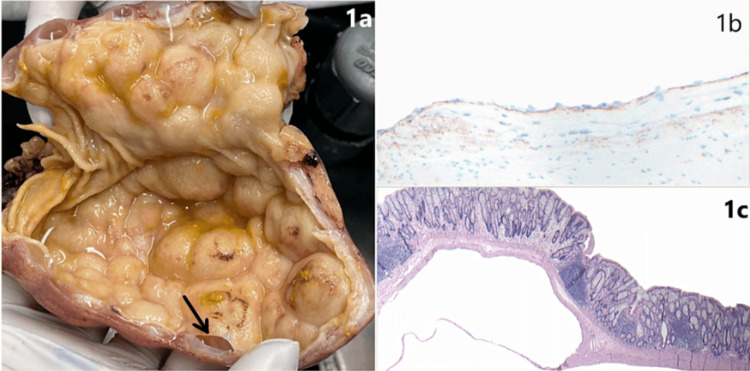
Colonic Lymphangiomatosis (1a) Gross image of the sigmoid colon, bottom aspect showing partial deroofing of the mucosa of a simple cystic cavity (black arrow). (1b) Hematoxylin and eosin (H&E) stain 40X, and (1c) D2-40 positivity (brown membranous and cytoplasmic staining) 100X.

The resected specimen showed large contiguous well-circumscribed and simple cystic structures (up to 3 cm in size) giving a sessile “polypoid” appearance of the mucosa (Figure 1a). Histologic sections demonstrated overlying unremarkable colonic mucosal surfaces without epithelial dysplasia or serrated neoplasia, large cystic spaces extending into the submucosa, focally separated by thin fibrous septae (Figure 1b). The contiguous cystic spaces within the muscularis propria were noted to be lined by a bland and attenuated cell layer, showing immunoreactivity for CD31 and D2-40 (podoplanin, an immunostain defining lymphatic space endothelium) (Figure 1c). The combined histologic and immunohistochemical findings were compatible with dilated lymphatic spaces. In conjunction with the reported mucosal abnormalities seen on the prior colonoscopic evaluation, the overall findings were consistent with the diagnosis of colonic lymphangiomatosis (CL). The patient had an uneventful postoperative course without complications, and outpatient follow-up colonoscopy one year after the surgery showed a healthy anastomosis in the sigmoid colon.

## Discussion

Lymphangiomas are rare benign tumors that are mostly incidentally found on colonoscopy, and most of them do not require resection [1]. As per the available literature, the likely etiology of CL has been suggested to range from developmental, or secondary to trauma, prior surgeries, radiation, lymphatic obstruction, and possibly secondary to adjacent mass lesions and/or previous inflammatory processes. Our patient did not have any of these risk factors. Lymphangiomatosis may affect other parts of the gastrointestinal tract as well. Depending on the size, location, and whether single or more diffuse, various symptoms have been reported in the literature prompting treatment in the form of endoscopic or surgical resection. CL has been associated with microcytic hypochromic anemia [1], protein-losing enteropathy [2], or acute presentations like adult colonic intussusception due to the lymphangioma serving as a leading point [3,4]. A case of lymphangiomatosis of the ileum complicated by perforation has also been reported [5].

Besides colonoscopy, imaging in the form of an abdominal CT scan, barium enemas, or endoscopic ultrasound has been used for the diagnosis [1,6]. Besides, imaging is also helpful in evaluating the nearby structures and excluding lymphangiomatosis of other parts like the mesocolon, omentum, or retroperitoneum [7]. Although endoscopic resection in the form of snare polypectomy or endoloop has been used for confirming the diagnosis and resection [1,8], when CL involves a large area in the colon, endoscopic resection may not be feasible. Surgical intervention is indicated in such symptomatic cases, especially ones that develop complications.

## Conclusions

In conclusion, CL should be considered as a differential in patients with multiple aggregated mucosal lesions that appear like polyps on endoscopy. Clinicians must have a high suspicion especially if such lesions are noted to be aggregated in a specific area of the colon. Free intraperitoneal air may suggest impending clinical decline in such patients which can be prevented by prompt diagnosis and clinical management.
